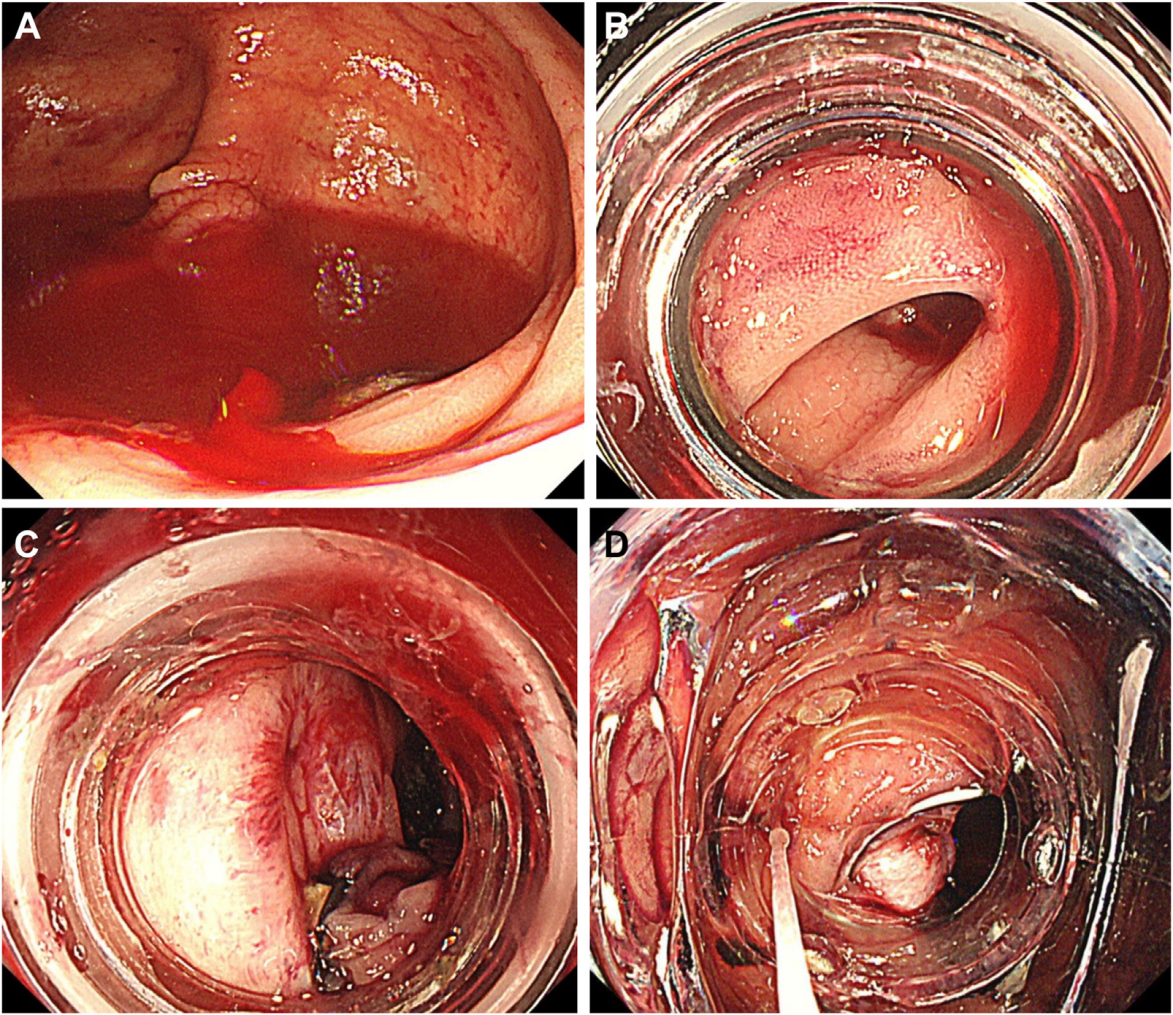# Over-the-Scope Clip as a Rescue Treatment for Colonic Diverticular Bleeding Abrased After Failed Endoscopic Band Ligation

**DOI:** 10.1016/j.gastha.2023.05.007

**Published:** 2023-06-07

**Authors:** Yuki Arai, Mimoe Konai, Naoki Ishii

**Affiliations:** Department of Gastroenterology, Tokyo Shinagawa Hospital, Tokyo, Japan

Endoscopic band ligation (EBL) is one of the first-line endoscopic treatments for colonic diverticular bleeding. However, when EBL is unsuccessful, the resulting incomplete suctioning and banding can lead to mucosal abrasion and difficulties with future endoscopic treatments. Over-the-scope clips (OTSCs) have recently been used to treat perforation, fistula, and refractory gastrointestinal bleeding. Here, we report on the application of OTSC as a rescue treatment for CBD after EBL.

An 80-year-old man who had been receiving hemodialysis and was regularly taking clopidogrel was referred due to acute hematochezia. Anal bleeding continued after admission. An urgent colonoscopy without a bowel purge was performed. Active bleeding was observed in the ascending diverticulum (Figure A), and an EBL was planned (Figure B). An O-band was released but was unsuccessful in banding the diverticulum, resulting in abrasion of the mucosa of and around the diverticulum (Figure C). An OTSC (blunt teeth type, Ovesco Endoscopy, Tübingen, Germany) was loaded onto the colonoscope. The OTSC was deployed, and the bleeding stopped (Figure D). There were no adverse events or recurrences of bleeding during the 18-month follow-up period. The OTSC was an effective rescue treatment for mucosal-abrased colonic diverticular bleeding after failed EBL.

**Figure:**